# Data on correlation among LAI, *T*/*R*, anthocyanin and starch content in purple fleshed sweetpotato during different growth stages

**DOI:** 10.1016/j.dib.2018.09.120

**Published:** 2018-10-03

**Authors:** Wei Tang, Mohamed Arisha, Yungang Zhang, Meng Kou, Hui Yan, Yaju Liu, Xin Wang, Daifu Ma, Qiang Li

**Affiliations:** a Xuzhou Institute of Agricultural Sciences in Jiangsu Xuhuai District / Key Laboratory for Biology and Genetic Breeding of Sweetpotato (Xuzhou), Ministry of Agriculture / Sweetpotato Research Institute, Chinese Academy of Agricultural Sciences, Kunpeng Road, Xuzhou, Jiangsu 221131, The People׳s Republic of China; b Faculty of Agriculture, Zagazig University, Egypt

## Abstract

Increasing demands for bio-products such as starch and anthocyanin stimulate researches towards purple-fleshed sweetpotato. Therefore, the aim of this complementary work is to identify the relationship between leaf area index, *T*/*R* value, starch and anthocyanin content in purple sweetpotato cultivar (cv “Xz3” from China) during the different growth stages. The traits were investigated every 15 days starting from 60 to 135 days after transplanting. The current data considered as a complementary for the main work “Quantifying cultivation technique and growth dynamics of purple-fleshed sweetpotato (*Ipomoea batatas* L.) in China” (Tang et al., 2018) [1].

**Specifications table**

**Table:** 

Subject area	Agricultural science
More specific subject area	Sweetpotato cultivation
Type of data	Figure and Table
How data was acquired	The leaves, stems and roots were sampled in the field and each trait was investigated by measurement and spectral analysis.
Data format	Statistically analyzed
Experimental factors	Correlation between different characters.
Experimental features	4 main traits including up-ground weight to storage root weight ratio (*T*/*R*) value, leaf area index (LAI), starch and anthocyanin content during the different growth stages of sweetpotato plants.
Data source location	Xuzhou Institute of Agricultural Sciences in Jiangsu Xuhuai District, Xuzhou, Jiangsu province, China
Data accessibility	Data is attached with this article
Related research article	W. Tang, Y.G. Zhang, Y.J. Liu, X. Wang, M. Kou, H. Yan, D.F. Ma, Q. Li, Quantifying cultivation technique and growth dynamics of purple-fleshed sweetpotato (*Ipomoea batatas* L.) in China, Field Crop Res. 227 (2018) 41–48.

**Value of the data**

• This data contribute in understanding and improving anthocyanin and starch content in purple sweetpotato cultivars.
• The data provide a method to elaborate main traits of sweetpotato which is available for use in sweetpotato breeding.

## 1 Data

**Fig. 1:**
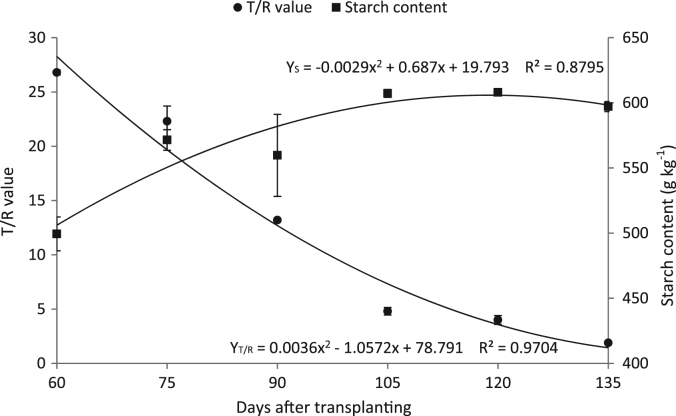
Changes of *T*/*R* value and starch content at six transplanting time.

This article presents data on the correlation between LAI, *T*/*R* value, starch and anthocyanin content in storage root on purple-fleshed sweetpotato cv “Xz3”during different growth stages. This data were investigated every 15 days from 60 to 135 DAT (day after transplanting). The data of correlations analysis among anthocyanin content, starch content, *T*/*R* value and LAI is shown in Table 1. Only *T*/*R* value has significant correlation with starch content and their dynamic changes are shown in Fig. 1.

**Table 1:** Correlations among anthocyanin content, starch content, *T*/*R* value and LAI.

	Anthocyanin content	Starch content	*T*/*R* value	LAI
Anthocyanin content	1	0.27	0.17	0.1
Starch content		1	-0.88**	0.27
*T*/*R* value			1	0
LAI				1

** The correlation is significant at *P* < 0.01.

## 2 Experimental design and materials

The experiment was conducted during the summer of 2014 in the Jiangsu Xuzhou Sweetpotato Research Center, Xuzhou City, Jiangsu Province (34 °16′N, 117 °17′E; 847 mm of annual rainfall). As complementary data for the main article, the new purple-fleshed sweetpotato cultivar, Xz3, which can be used as a source for anthocyanin pigment, was selected for the present study. In order to investigate the effect of LAI and *T*/*R* value on starch and anthocyanin content in Xz3 sweetpotato cultivar. The seedlings were transplanted to the field and grew based on its optimum cultivation practices [1]. Five randomly samples were collected at 60, 75, 90, 105, 120, and 135 DAT, respectively to determine the previously mentioned traits.

## 3 Methods

LAI was measured according to the direct harvesting method [2]. Anthocyanin content was determined via citric acid-disodium hydrogen phosphate buffer extraction [3]. The contents of starch was determined by VECTOR22/N-type Fourier transform near-infrared reflectance spectroscopy (Bruker Optics, Germany) [4].

## 4 Data analysis

Statistical analysis of collected data was performed using Excel 2010 and SPSS (IBM, USA), and the significance level was adopted for multiple comparisons (*P*< 0.05, 0.01). Quadratic equations were used to estimate the dynamic changes of *T*/*R* value and starch content in Xz3. The best models were determined based on the overall highest coefficient of determination (*R*^2^) and the least root mean square error (RMSE) values.
