# Supplementary material for: Data on correlation among LAI, T/R, anthocyanin and starch content in purple fleshed sweetpotato during different growth stages
Source: Data Brief. 2018 Oct 3;21:88–91. doi: 10.1016/j.dib.2018.09.120 (PMC6187010; doi:10.1016/j.dib.2018.09.120)
Supplement: Supplementary file 1 — Supplementary material [file mmc1.docx]

Conflict of Interest

The authors declare that they have no conflict of interest
